# Supplementary figures and images for: Development and internal validation of a nine-lncRNA prognostic signature for prediction of overall survival in colorectal cancer patients
Source: PeerJ. 2018 Dec 6;6:e6061. doi: 10.7717/peerj.6061 (PMC6286799; doi:10.7717/peerj.6061)

# Volcano

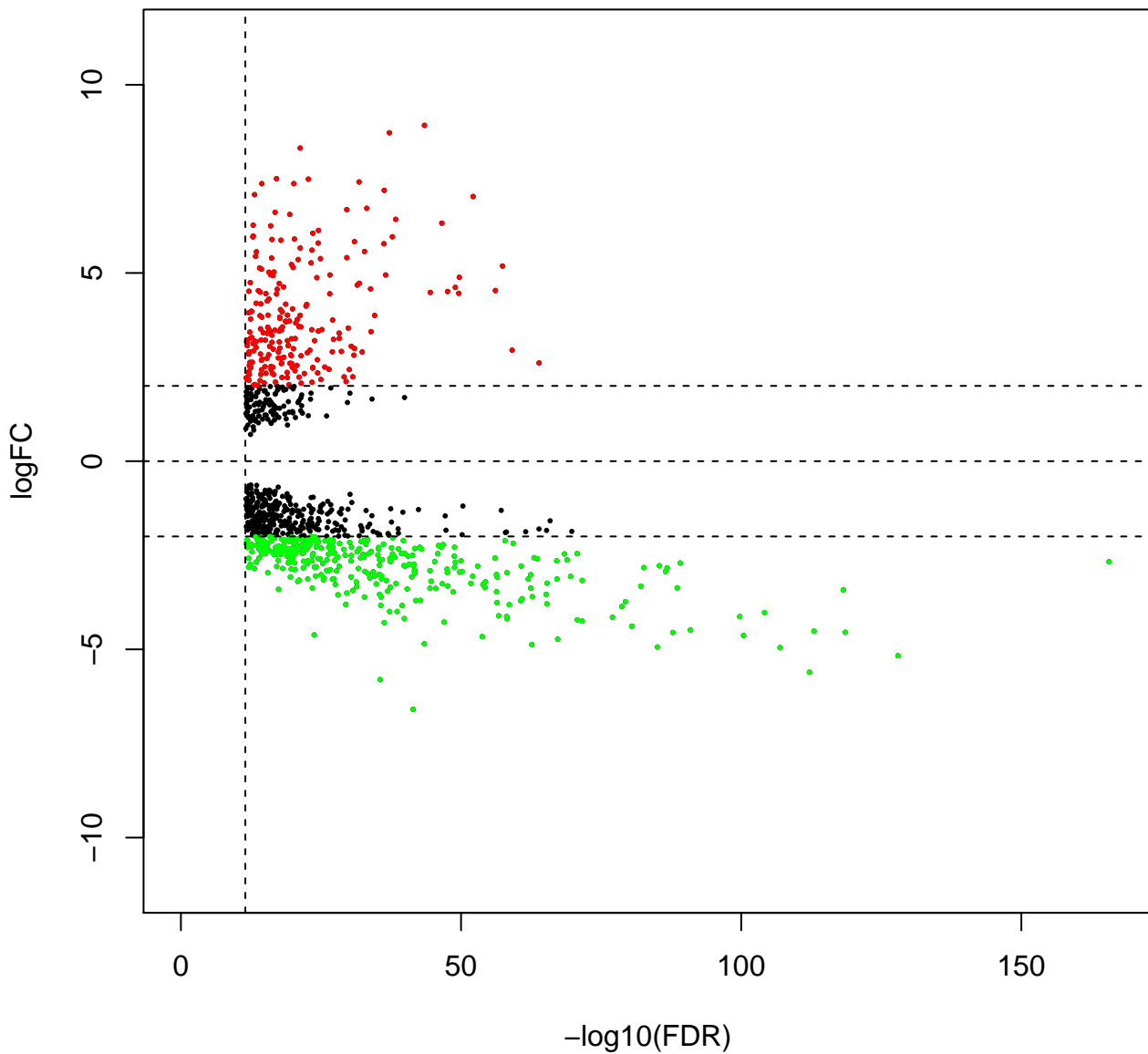

Supplement: Figure S2 [file peerj-06-6061-s002.pdf]
