# Supplementary material for: Development and internal validation of a nine-lncRNA prognostic signature for prediction of overall survival in colorectal cancer patients
Source: PeerJ. 2018 Dec 6;6:e6061. doi: 10.7717/peerj.6061 (PMC6286799; doi:10.7717/peerj.6061)
Supplement: Figure S1 [file peerj-06-6061-s001.pdf]

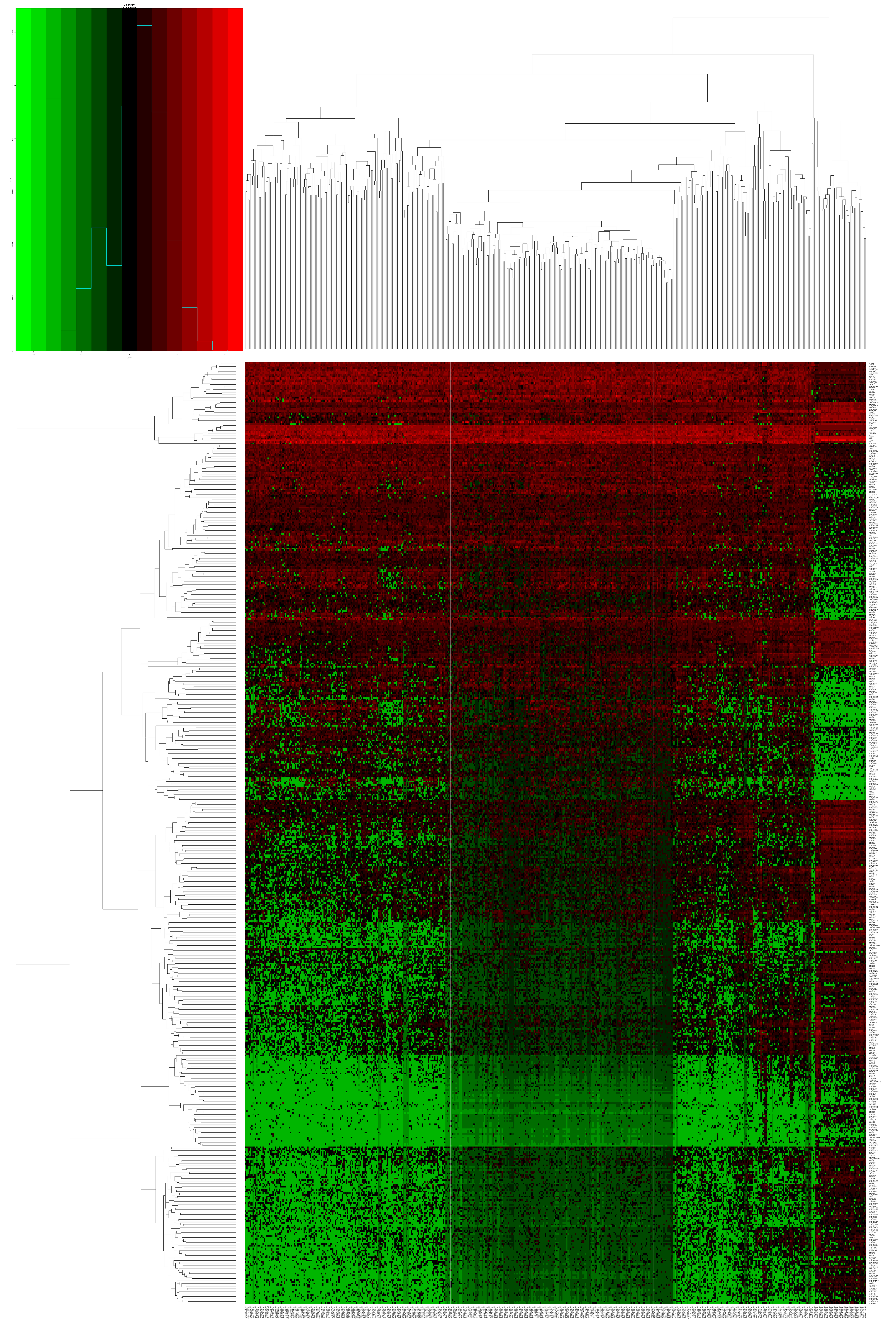
Color Key
and Histogram
Count
60000
50000
40000
30000
20000
10000
0
−4
−2
0
2
4
Value
